# Grammatical Encoding in Bilingual Language Production: A Focus on Code-switching

**DOI:** 10.3389/fpsyg.2015.01797

**Published:** 2015-11-26

**Authors:** Mehdi Purmohammad

**Affiliations:** Center for the Study of Language and Society (CSLS), University of BernBern, Switzerland

**Keywords:** bilingualism, bilingual language production, code-switching, grammatical encoding, syntactic processing

## Abstract

In this study, I report three experiments that examined whether words from one language of bilinguals can use the syntactic features form the other language, and how such syntactic co-activation might influence syntactic processing. In other words, I examined whether there are any cases in which an inherent syntactic feature a lexical item is inhibited and the syntactic feature that belongs to the other language is used, instead. In the non-switch condition in Experiments 1 and 2, Persian-English bilinguals described pictures using an adjective–noun string from the same language requested. In the switch condition, they used a noun and an adjective from the other language. In the switch condition in Experiment 3, participants used only the adjective of a noun phrase from the other language. The results showed that bilinguals may inhibit the activation of a word’s syntactic feature and use the syntactic property from the other language, instead [e.g., pirāhane (shirt-N) black]. As the combinatorial node (the node that specifies different kinds of syntactic structures in which a word can be used) of a used adjective retains activation at least temporarily, bilinguals are more likely to use the same combinatorial node even with an adjective from the other language. Cross-language syntactic interference increased in the switch conditions. Moreover, more inappropriate responses were observed when switching from bilinguals’ L2 to L1. The results also revealed that different experimental contexts may lead to different patterns of the control mechanism. The results will be interpreted in terms of Hartsuiker and Pickering’s (2008) model of syntactic representation.

## Introduction

Code-switching (CS) is defined as a change from one language of a bilingual speaker to another in the same utterance or conversation (Hamers and Blanc, 1989). CS is a common language phenomenon that occurs in bilinguals’ speech production. Example (1) shows CS between English and Spanish:

(1) Dónde está ese paño blue?

‘Where is that blue cloth?’ Arias and Lakshmanan (2005, p. 104)

The CS phenomenon has been widely discussed in a variety of fields. In comparison with all other contact phenomena of interest, CS “has arguably dominated the field” (Bullock and Toribio, 2009, p. 1). Psycholinguistic research on aspects of bilingual language production has focused on general modeling issues (e.g., de Bot, 1992; de Bot and Schreuder, 1993), the control of processing (e.g., Green, 1993, 1998), and the formulation of output (e.g., Myers-Scotton, 1993, 2002) (Karousou-Fokas and Garman, 2001). In all approaches, the CS data are viewed as important sources of evidence. Studies on CS can help psycholinguists, for instance, find whether one of the two languages is deactivated while the other language is being activated, and “how incoming signals are channeled to their appropriate decoding system for interpretation (e.g., input switch)” (Paradis, 1993, p. 135).

Code-switching in constructions containing an adjective has received a lot of attention in structural linguistics. Most structural approaches to CS look for formulating some constraints on CS. For about three decades now, the main aim of positing the constraints has been to formulate the interaction between the two grammars of a bilingual speaker in CS (Mahootian, 2006). Some earlier research (e.g., Pfaff, 1979; Sankoff and Poplack, 1981) proposed that CS is not allowed at points where the two languages in contact do not share the same word order representation (see MacSwan, 2009). Accordingly, “adjective/noun mixes must match the surface word order of both the language of the adjective and the language of the head noun” (Pfaff, 1979, p. 306). In this view, since Persian and English do not share the same adjective–noun order, switching inside NPs is prohibited. Some researchers (e.g., Aguirre, 1976; McClure, 1977, 1981) argued that switching inside NPs is possible so long as the placement rule of the adjective language is met. The Equivalence Constraint Model of Code-switching (Poplack, 1980; Sankoff and Poplack, 1981) stipulates that language switching tends to occur at points where the two languages have the same word order representation. Thus, according to Poplack (1980) and Sankoff and Poplack (1981), since the syntactic rule of one of the two languages is violated in the Persian-English switches inside the NP structures, switches do not occur.

Purmohammad (2015) investigated the grammatical encoding in code-switched utterances. He collected 2293 min of a popular TV show. Persian-English bilinguals freely inserted English words into their Persian utterances. 962 code-switched utterances were found. He reports that 210 switched words were adjectives. In 10% of the cases, Persian-English bilinguals used English adjectives after the Persian nouns.

Cantone and MacSwan (2009) investigated how linguistic properties relevant to determining surface word order for adjectival constructions are resolved in CS contexts in which languages with different word order are involved. In line with this, 10 participants gave their grammaticality judgments for the mixed utterances involving determiners, adjectives, and nouns (e.g., in un Bett nuovo meaning a bed new) by determining whether each utterance was well-formed or not. The results of the study showed that whenever the language of the adjective was reflected in the word order of the mixed utterances, participants judged them to be acceptable; whereas those mixed utterances in which the language of adjective was not matched were judged to be ill-formed. Some researchers (e.g., Pandit, 1990) assumed that the language of the head noun determines the syntactic properties of its complements; however, Nartey (1982, cited in Cantone and MacSwan, 2009) assumed that in the Adanme-English CS, the Adanme determiner determines noun-adjective order. Belazi et al. (1994) claim that the language of the adjective determines the adjective–noun order. As we will see later, the results of the present study are inconsistent with the constraints proposed on adjective–noun switches; however, we will not go into more details here (see Gil et al., 2012 for more discussion).

Bilingual speakers know two different languages and hence they know two different grammatical systems. For example, one of the two languages of a Persian-English bilingual speaker uses post-nominal adjectives (adjectives follow nouns) whereas the other language (English) uses prenominal adjectives (adjectives precede nouns). Although ample evidence has led researchers to assume that the two languages are co-activated during lexical processing, a fundamental question is whether the parallel activation of the two languages leads to interference (Hatzidaki et al., 2011). One group of researchers assumes that although the two languages of bilinguals are activated during sentence production, the non-target language does not affect the target language. For example, La Heij (2005) proposed that the intended language acts as a language cue. It ensures that lexical items in the intended language reach a higher activation level than their equivalent translations in the non-intended language. The second group of researchers suggests that activation of the non-intended language can influence lexical processing in the target-language (see Costa, 2005 for review). For example, Costa et al. (2006) tested for the lexical bias effect (LBE). This effect shows “feedback between the phonological and lexical levels of representation during speech production” (p. 972). The LBEs suggest that feedback existing in second-language production extends across the two languages of a bilingual speaker. They conclude that representations of both languages are recruited in bilingual language processing even when only one language is used.

As stated above, there is compelling evidence (e.g., Francis, 2005; Kroll et al., 2006, 2008; Voga and Grainger, 2007) indicating that aspects of the two languages of bilinguals are activated during both unilingual and bilingual modes (see Grosjean, 2008 for language mode account). Thus, we expect syntactic interference from the non-target language. Although the results of studies has provided the researchers the evidence to assume that components of the two languages (e.g., syntax, phonology) are activated during language processing, it remains contentious what exactly means by interference, for instance, the syntactic interference, especially from a processing perspective. More importantly, it remains unclear how the processor operates during language interference. For example, what language processing mechanism underlies the sentence in which a bilingual uses a prenominal adjective (e.g., Spanish “chiquita” meaning small) post-nominally? (see example 2).

(2) I went to the house *CHIQUITA*.

I went to the little house. (Pfaff, 1979, p. 307)

This study examines whether words from language A can use the syntactic features form language B and how such syntactic co-activation might influence syntactic processing. To put it differently, the main aim is to examine whether there are any cases in which an inherent syntactic feature (e.g., post-nominality) of a lexical item (e.g., an adjective) is inhibited and the syntactic feature that belongs to the other language is used, instead. If this were the case, how such linguistic behavior could be captured within a model of bilingual language production.

The present study reports three experiments that investigate the processing of adjective–noun strings in code-switched utterances. More specifically, I examine how the activation of adjective placement rule from the non-target language may affect the syntactic processing of the structures containing a noun and an adjective. In all three experiments, participants use adjective–noun strings in order to name pictures. If their language productions differ with respect to using syntactic features in three experiments, I will discuss what factors might cause such differences. If the grammatical features of the non-target language affect the target language, for instance, using an English adjective post-nominally (e.g., “ketābe different” lit. “book different”), this would give evidence to suggest that bilingual’s two language systems interfere during language processing. Finally, I examine how the results of the present study might be integrated with Hartsuiker and Pickering’s (2008) integrated model of syntactic representation.

According to Hartsuiker and Pickering’s (2008) model, bilingual speakers have an integrated lemma stratum. It is assumed that lemmas – the base form of each word- from the two languages are represented in an integrated network. Each lemma node (e.g., red in English or *qermz* in Persian) is linked to one conceptual node [RED(X,Y)] at the conceptual stratum, to one category node (e.g., adjective, noun), to combinatorial nodes (e.g., prenominal or post-nominal adjective), and to one language node (e.g., English, Persian) in their integrated network. In this model, category nodes specify grammatical categories (e.g., adjective) and combinatorial nodes specify different kinds of syntactic structures in which a word can be used (Bernolet et al., 2007). One of the important aspects of the model is that featural, combinatorial, and category nodes are shared in a way that reduce redundancy (Cleland and Pickering, 2003). Accordingly, the lemma nodes such as “nice” and “brown” are both linked to the same category node (adjective) and combinatorial node (prenominal).

Cross-linguistic grammatical effects and lexical switching are predicted in this model, because in this model both meaning and syntax of lexical items are points of contact across languages (Hartsuiker et al., 2004). Thus, according to the model’s prediction it is possible that a Persian-English bilingual speaker selects a Persian construction (e.g., a noun-adjective word order string) when using an English adjective (e.g., a book red). However, no effect of language proficiency on cross-linguistic influences was predicted by the model (Hartsuiker and Pickering, 2008).

Given that Persian uses adjectives post-nominally while English generally uses adjectives prenominally, it seems that adjective placement is suitable for the purpose of the study, because the results may better show how the syntactic components of the two languages of bilinguals interact during speech production compared to the situation in which both languages use the same adjective placement rules. In this study, adjectives are used either prenominally or post-nominally in their corresponding languages. When interference occurs, an adjective is likely to cede its combinatorial feature (prenominal or post-nominal) to the other combinatorial feature. A model of bilingual syntactic representation needs to explain how the production of a lexical element is influenced by the syntactic properties of the other language.

Investigating syntactic interference is crucial because this phenomenon permits us to know how the grammars of the two languages are represented in bilinguals’ memory; how the grammars of the two languages interact during production; how grammatical functions are assigned to concepts, and more importantly how the mental lexicon and syntactic encoding interface in bilingualism (Hartsuiker and Pickering, 2008). All three experiments reported in this study include switching tasks. Since the aim of the study is to test whether there are any cases in which bilingual speakers use the grammar of one language and the words from the other language, it seems that language switching tasks are suitable for the purpose of the study, because when a bilingual speaker switches between the two languages, he or she has to consider using two different grammatical systems in a single utterance.

## Experiments

The present study consists of three experiments. In all experiments a picture-naming task was used. In each trial, participants were presented with a sentence fragment along with a picture depicted above the sentence fragment. In Experiment 1, in the non-switch conditions, participants described pictures using an adjective–noun string from the language of the sentence fragment. In the switch conditions, however, they completed the sentence fragments using a noun and an adjective from the other language (see **Table 2**, for sample items used in Experiment 1). In Experiment 2 in the switch conditions, participants were presented with a sentence fragment in language A along with a picture depicted above it. A noun phrase including q noun and an adjective from language B was printed above the target picture as well (see **Table 5**, for sample items used in Experiment 2 and Appendix A for the items used in Experiment 2). Participants had to use the translation-equivalents of the noun phrase in order to describe pictures. In the non-switch conditions, however, they used both nouns and adjectives from the language of the sentence fragment. In each trial in Experiment 3, participants were presented with a sentence fragment along with a picture depicted above it. In the switch conditions, participants used only the adjectives of noun phrases from the other language; however, they used the noun from the language of the sentence fragment. In the non-switch conditions, they had to use a noun and an adjective from the language of the sentence fragment (see Appendix B for the items used in Experiment 3).

All experiments consisted of two main conditions (the switch and non-switch conditions) and four different sets of items: the Persian set, the Persian-English set, the English set and the English-Persian set. The Persian and English sets of items represented the non-switch conditions and the Persian-English and English-Persian sets of items represented the switch conditions. The experiments, thus, had a 2x2 experimental design for language task (the switch vs. non-switch condition) and language (Persian vs. English; Persian-English vs. English-Persian).

Consistent with Hatzidaki et al. (2011), it is hypothesized that since the two languages of bilingual speakers are activated during language production, the grammatical system of the non-target language may affect the production of the target language. Moreover, it is hypothesized that more inappropriate responses in which a word from language A uses a syntactic feature from language B (e.g., “marde tall” lit. “man tall”) are made in the tasks that involve switching (i.e., in bilingual contexts) than in the unilingual contexts involving no switching, because in the switch conditions the two languages of a bilingual speaker must inevitably be activated and that in the switch conditions both languages are activated to a greater degree compared to the non-switch conditions.

## Experiment 1: Sentence Completion Task 1

### Method

#### Participants

Thirty six Persian (L1)-English (L2) bilinguals took part in the experiment. Participants were recruited through advertisements which clearly stated proficiency in both Persian and English as prerequisite. They were paid six pounds for their participation. Eighteen of them were Ph.D. students at Heriot-Watt University or the University of Edinburgh. Eleven participants hold master’s degrees from the UK universities. Two of them were university professors. Five participants were high school students in Edinburgh. They all reported having normal vision. Their self-ratings of English language skills (speaking and listening) and the results of the English proficiency test demonstrated that the participants were fluent in English. The median age of the participants was 30.5 years with a median length of residence of 8 years in UK. **Table 1** shows the participants’ background characteristics in all three experiments reported in this study.

**Table 1:** Participants’ characteristics in Experiments 1–3.

	EXP 1	EXP 2	EXP 3
Measures	*N* = 36	*N* = 37	*N* = 29
Age Self-rated speaking ability in English (seven-point scale) Self-rated listening ability in English (seven-point scale) English language proficiency test mark (the highest score: 25) Years of English language use in daily life Self-reported amount of code-switching (five-point scale)	30.5 5.55 5.69 21.75 8.62 2.52	29.72 5.59 5.72 21.86 8.87 2.53	29.20 5.55 5.62 22.06 8.77 2.61

*EXP: Experiment, N: number of participants.*

#### Materials

Thirty-two sentence fragments were created. The 32 sentence fragments included eight items from the Persian set, the English set, the Persian-English set, and the English-Persian set. In each trial, the name of a common object was omitted. Thirty-two unique pictures were presented in the place of the omitted objects. For the Persian set, the green outlined pictures were used to satisfy Persian as the response language. Then a mixture of eight green outlined pictures with eight Persian sentence fragments was used for the Persian set. For the English set, the orange outlined pictures were used to satisfy English as the response language. A mixture of eight orange outlined pictures with eight English sentence fragments was used for the English set. The English-Persian set was created by combining the English sentence fragments with the green outlined pictures. The Persian-English set was created by combining the Persian sentence fragments with the orange outlined pictures. In each experiment, 32 highly frequent nouns (16 nouns for the English set and 16 nouns for the Persian set) and 32 highly frequent adjectives (16 adjectives for the English set and 16 adjectives for the Persian set) were used. It is common to use background-color-cueing procedure in language switching studies (see Meuter and Allport, 1999; Costa and Santesteban, 2004; Kootstra et al., 2010; Broersma, 2011).

Two randomized versions of the same presentation list were constructed. Each list included 32 items. Sixteen Persian sentence fragments were constructed and their English translations were used for the English set. A group of five Persian-English speakers was asked to check for the accuracy of English sentences. Pictures were identical in all sets. Sixteen Persian-English sentence fragments were provided and their English translations were used for the English-Persian set. Each list contained eight items from each set (the Persian set, the English set, the Persian-English set, and the English-Persian set). Then Experiment 1 included 16 switch conditions and 16 non-switch conditions. **Table 2** shows sample items used in Experiment 1.

**Table 2:** Sample items used in Experiment 1.

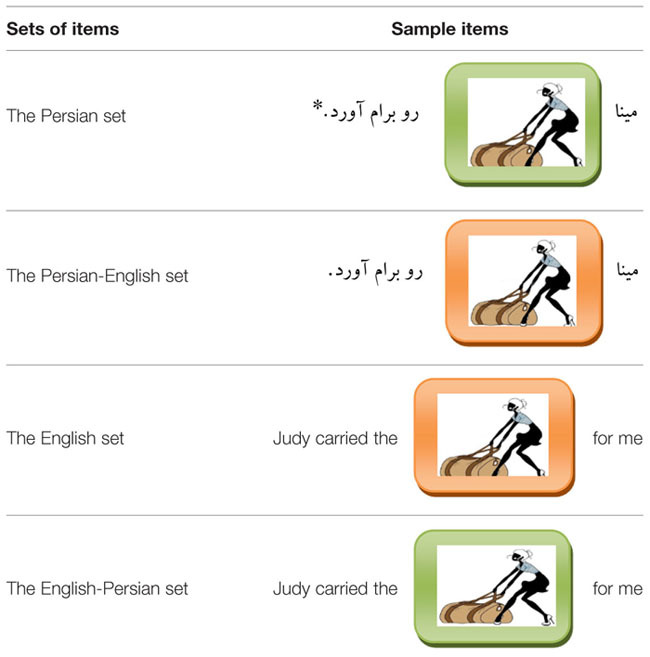

The table shows the basic design used in Experiment 1. Two semantically identical items were not used in a single list. ^∗^Mina carried the “heavy bag” for me.

Since the English sentence fragments were the translations of Persian sentence fragments, each list was designed so that participants did not receive two semantically identical items. Trials were in randomized order.

There is a concern that different classes of adjectives may work differently (Sobin, 1984). In the present study, different types of adjectives (e.g., color, feeling, appearance, shape, size) were used; however, Sobin (1984) used color adjectives only.

#### Procedure

Before doing the experiments, participants were asked some demographic questions including name, age, sex, and the number of the years they used English in their daily life. Prior to the experiments, participants were given four practice trials in order to familiarize themselves with the experimental tasks. Instructions were given in Persian. Participants were informed that their speech would be recorded. Each participant was tested individually. They sat in front of the same laptop and completed the sentence fragments.

In each trial, a sentence fragment along with a picture depicted above it was presented to the participants. Participants were instructed to read the entire sentence fragment out loud and to fill in the missing part. In order to describe the pictures presented in the place of the omitted objects (see **Table 2** for sample items used in Experiment 1), they had to use a noun and an adjective. By doing so, they completed 32 sentence fragments. While the green outlined pictures showed that Persian should be the response language, the orange outlined pictures showed that English should be the response language. Therefore, in the non-switch conditions, if the sentence fragments were in Persian and the pictures had a green background color, participants had to use a Persian noun and an adjective to complete the sentence fragments. In the switch conditions, when the sentence fragments were in Persian and the pictures had an orange background color, they had to use an English noun and an adjective to complete them. In the same way, if the sentence fragments were in English and the pictures had a green background color, participants had to use a Persian noun and an adjective to complete the sentence fragments. They were told that there was no preferable way of doing the task.

A 25-items cloze test was constructed to rate participants’ English language proficiency. Participants were instructed to fill in the blanks with the most appropriate English words.

#### Scoring and Data Analysis

Three different categories were used to score participants’ responses. Responses were scored as “appropriate” when participants completed the sentence fragments as requested (i.e., using an English adjective prenominally and using a Persian adjective post-nominally). Responses were scored as “inappropriate” when they did not complete the sentence fragments as requested. Then a response that used an English adjective post-nominally (e.g., “chiz-e different” lit. “thing different”) is considered as an “inappropriate” response. Responses were scored as “other” for all other completions. For example, if participants failed to complete a sentence fragment, it would be scored as “other.” Moreover, all responses had to use a noun-adjective string only. All other strings (e.g., a lot of books) were scored as “other” and omitted from the analyses.

The scoring criteria need more clarification. Responses were scored as “appropriate” when participants used the correct adjective placement rule of the language that the adjective belongs to (e.g., “tall mard” lit. “tall man”). Accordingly, prenominality is considered as an inherent feature of adjectives in English and post-nominality is considered as an inherent feature of adjectives in Persian. Then a response that used an English adjective post-nominally (e.g., “chiz-e different” lit. “thing different”) is considered as “inappropriate.” Note that I did not consider the structural accounts on the syntactic structure of CS involving adjectival constructions, because all the responses in the switch conditions are inconsistent with the structural accounts in which language switching is not allowed at points where the two languages do not share the same word order representation (see Gil et al., 2012). Moreover, there is ample evidence indicating that neither the head noun nor the adjective, nor the language of the determiner (e.g., a, the) determine the adjective–noun order (see Introduction; Cantone and MacSwan, 2009 for review). I am concerned whether or not adjectives use the adjective placement rule of the language they belong to. Thus, “appropriateness” here does not mean that participants used the correct adjective–noun order in language A or B, because when the two languages of bilinguals use different adjective–noun ordering, we always expect that the switch containing a noun and an adjective does not respect the language-specific requirement of one of the two languages involved.

Similar to Hatzidaki et al. (2011) and Selles (2011), a linear mixed effect was used to test whether the inappropriate responses were affected by language task (the switch and non-switch conditions), language proficiency, source language, target language, and participants’ self-ratings of their speaking and listening skills. Using appropriate and inappropriate responses as the dependent variables and experimental items and participants as random effects, first a null model was created. To find the model with the best fit, predictors were added to the model individually. Then using χ^2^-tests, the models were compared to see whether adding the predictors contributed significantly to the model.

### Results

Overall, 1152 sentence fragments including 576 switched and 576 non-switch utterances were completed by the participants. There were 10 (0.86%) “other” responses and removed from the analyses. The analysis is based on the remaining 1142 sentence fragment completions. The results of Experiment 1 showed that appropriate responses occurred more frequently (98%) than inappropriate responses (2%). The number of appropriate responses was almost the same in the non-switch conditions (98%) and in the switch conditions (97%). Moreover, the results demonstrated that inappropriate responses occurred more frequently in the switches from L2 to L1 (78.52%) than from L1 to L2 (21.42%). **Table 3** reports the participants’ responses per condition.

**Table 3:** Experiment 1: participants’ responses in the switch and non-switch tasks.

	Responses			
Language task	Sum	Appropriate	% inappropriate	Omission
Non-switch tasks	576	570	4 (0.70)	2
Persian	288	283	4 (100)	1
English	288	287	0 (0.00)	1
Switch tasks	576	554	14 (2.43)	8
Persian-English	288	280	3 (21.42)	5
English-Persian	288	274	11 (78.57)	3

*LT, Language task; Omission: responses scored as other, % inappropriate: the percentage of inappropriate responses (responses scored as other were not included).*

Using a linear mixed effect model, a baseline model was created using participants and items as random effects. The logistic variant was used. Items and participants were used as random slopes. I incrementally added predictors to the base line model and χ^2^-tests were conducted to determine which of the predictors attributed to the model of best fit (see **Table 4**). Language task, target language, and source language were tested individually as predictors. Language task and target language were individually significant but source language was not significant. Finally, both language task and target language were added to the base model as predictors and the results were highly significant. χ^2^-tests showed that the model of best fit used language task and target language as predictors.

**Table 4:** Models of responses in Experiment 1.

Predictor	Estimate	*SE*	*z*-value	*p*
Language task as main predictor: χ^2^(1) = 5.618, *p* = 0.018, *N* = 1142				
(Intercept)	-10.445	1.727	-6.049	<0.001
Language task	1.479	0.685	2.159	0.031
Target language as main predictor: χ^2^(1) = 8.578, *p* = 0.003, *N* = 1142				
(Intercept)	-5.646	1.536	-3.675	<0.001
Target language	-1.846	0.723	-2.552	0.011
Language task and target language as predictors: χ^2^(1) = 15.601, *p* < 0.001, *N* = 1142				
(Intercept)	-8.284	1.938	-4.275	<0.001
Language task	1.508	0.642	2.349	0.019
Target language	-1.899	0.706	-2.689	0.007

As the language task variable is a combination of the two other variables (source language and target language), it may be redundant to include it as a predictor. Thus, it would be sufficient to consider only source language and target language as predictors. Dropping language task from the data analysis yields the following results: no significance in target language × source language interaction, and a main effect of target language (*p* < 0.003). The results also indicated that the language × condition (the switch/non-switch condition) interaction was not significant (*p* > 0.7).

To test to see whether language proficiency put an effect on responses, further predictors were added based on the rating of participants’ proficiency levels. English proficiency tested in interaction with experimental predictors yielded the following results: no significance of self-rated language proficiency × target language, language proficiency × source language, self-rated speaking proficiency × target language, self-rated speaking proficiency × source language, self-rated listening proficiency × target language, or self-rated listening proficiency × source language (see **Table 1** for different measures of language proficiency level).

### Discussion

In sum, the results of Experiment 1 demonstrated that the adjective–noun order of the intended language was a strong predictor of participants’ performance both in the switch and non-switch conditions. However, the results revealed that as both languages of bilingual speakers were co-activated, participants showed interference from the non-target language on the target language. Responses were not affected by participants’ levels of language proficiency.

## Experiment 2: Sentence Completion Task 2

To get a better picture of the nature of the syntactic processing in code-switched utterances, an additional sentence completion task (Experiment 2) was designed. Experiment 2 investigates whether similar results would occur with a different task in which participants use the translation equivalents of the noun phrases printed above pictures in the switch conditions.

### Method

#### Participants

Thirty-seven participants took part in Experiment 2. Thirty-six of them were from the same population as Experiment 1. **Table 1** demonstrates the participants’ characteristics.

#### Materials

Thirty-two sentence fragments were created. Thirty-two unique pictures were presented in the place of the omitted objects (see Appendix A for the items used in the experiment). The pictures were identical across the four language sets. The main difference between the switch and non-switch trials was that in the switch conditions noun phrases from the base language (the language of the sentence fragments) were printed above the target pictures. Participants had to use the translation-equivalents of the noun phrases printed above the target pictures. But in the non-switch trials, they had to use a noun and an adjective from the base language to describe pictures. As in Experiment 1, the 32 sentence fragments included eight items from the Persian set, the English set, the Persian-English set, and the English-Persian set. Then Experiment 2 consisted of 16 switch trials and 16 non-switch trials. **Table 5** shows sample items used in Experiment 2.

**Table 5:** Sample items used in Experiment 2.

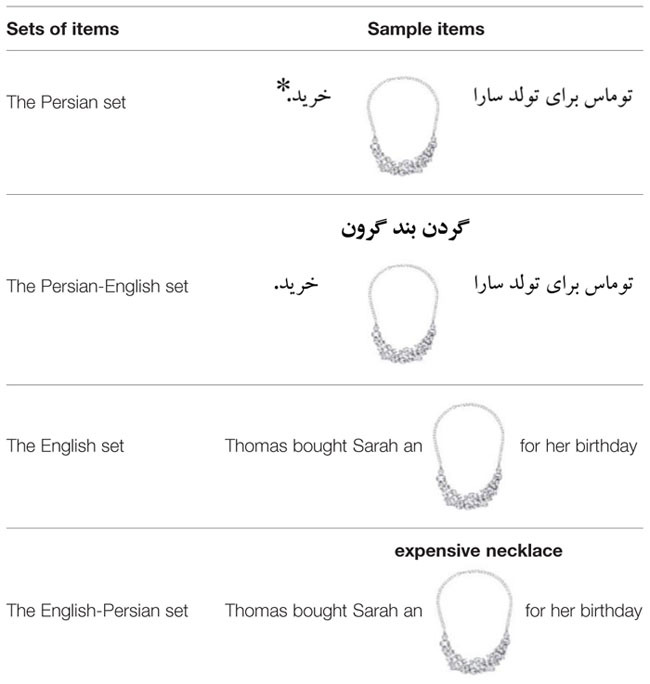

*The Table shows the basic design used in Experiment 2. Two semantically identical items were not used in a single list. ^∗^Thomas bought Sarah an “expensive necklace” for her birthday.*

Two randomized versions of the same presentation list were constructed. Since the English sentence fragments were the translations of the Persian sentence fragments, the lists were arranged so that not each participant received two semantically identical items.

#### Procedure

Participants were instructed that in the switch conditions they would first read the noun phrases printed above the target pictures. To describe pictures, they had to use the translation-equivalents of the noun phrases printed above the target pictures. Participants were told that in the non-switch trials, they had to use a noun and an adjective from the language of the sentence fragment (base language). Prior to the experiments, participants were given four practice trials in order to familiarize themselves with the experimental tasks. Participants were informed that their speech would be recorded. They were instructed entirely in Persian.

#### Scoring and Data Analysis

The scoring and data analysis were identical to those described in Experiment 1.

### Results

Overall, 1185 sentence fragments consisting of 592 switched and 592 non-switched utterances were completed by the participants. There were 10 (0.84%) “other” responses and discarded from the analysis. The following analysis is based on the remaining 1175 responses. The results showed that the global pattern of responses was identical to those in Experiment 1. Similar to Experiment 1, in most cases (98%) participants used the correct adjective placement rules. Participants produced more appropriate responses (99%) in the non-switch conditions than in the switch conditions (96%). **Table 6** shows participants’ responses per condition. The results also revealed that inappropriate responses occurred more frequently in switches from L2 to L1 (89.47%) than in switches from L1 to L2 (10.52%).

**Table 6:** Experiment 2: participants’ responses in the switch and non-switch tasks.

	Responses			
Language task	Sum	Appropriate	% inappropriate	Omission
Non-switch tasks	592	582	1 (0.16)	9
Persian	296	292	0 (0.00)	4
English	296	290	1 (100)	5
Switch tasks	592	572	19 (3.20)	1
Persian-English	296	293	2 (10.52)	1
English-Persian	296	279	17 (89.47)	0

*LT, Language task; Omission: responses scored as other, % inappropriate: the percentage of inappropriate responses (responses scored as other were not included).*

The results were calculated as described in Experiment 1. Target language and source language were individually significant, but language task (trial type) was not. When target language and source language were both added as predictors they had significant effects on model. As in Experiment 1, χ^2^-tests were conducted to determine the model of best fit (see **Table 7**). With the χ^2^-tests, it was found that the model with source language and target language as predictors was the model of best fit. Dropping language task from the data analysis did not change the results. There was a main effect of target language and source language (*p* < 0.005). Having removed language task as a predictor, target language is the model of best fit. The results revealed that the language × condition interaction was not significant (*p* > 0.1). Language proficiency was tested in interaction with the experimental predictors. Similar to Experiment 1, neither language proficiency, nor self-rating of speaking skill, nor self-rating of listening skill improved the model.

**Table 7:** Models of responses in Experiment 2.

Predictor	Estimate	*SE*	*z*-value	*p*
Target language as main predictor: χ^2^(1) = 7.995, *p* = 0.005, *N* = 1174				
(Intercept)	-5.106	1.807	-2.826	0.005
Target language	-2.680	1.148	-2.336	0.020
Source language as main predictor: χ^2^(1) = 7.997, *p* = 0.005, *N* = 1174				
(Intercept)	-13.145	2.431	-5.406	<0.001
Source language	2.680	1.147	2.336	0.020
Source language and target language as predictors: χ^2^(1) = 19.318, *p* < 0.001, *N* = 1174				
(Intercept)	-9.009	2.127	-4.237	<0.001
Source language	2.568	0.838	3.064	0.002
Target language	-2.567	0.838	-3.064	0.002

### Discussion

The purpose of Experiment 2 was to examine whether a different language task (a translation task) would yield different responses. In the switch trials participants used the translation-equivalents of the noun phrases printed above pictures in order to describe the target pictures. In the non-switch trials; however, they used a noun and an adjective from the language of the sentence fragments. The results showed that as in Experiment 1, in most cases participants used the Persian adjectives post-nominally and the English adjectives prenominally. The results, however, revealed that the intrinsic syntactic feature (the prenominal or post-nominal features) of an adjective can be inhibited and the syntactic feature from the other language can be used, instead. Inappropriate responses were not affected by participants’ levels of language proficiency.

Experiment 2 is important because in this experiment, again participants had to use a noun and an adjective from the base language (the non-switch condition) or from the other language (the switch condition). What the results may suggest above and beyond Experiment 1 is that when both the noun and adjective are from the same language, adjectives were appropriately located. The results show that the context or the task in which a word is produced may affect the syntactic processing during sentence production.

## Experiment 3: Sentence Completion Task 3

Experiment 3 examines whether using the syntactic features (combinatorial nodes) from the other language enhances when only adjectives from the other language have to be used in the switch conditions. Experiment 3 used the same design as Experiment 1 except that in the switch conditions participants used only adjectives from the other language.

### Method

#### Participants

Twenty-nine subjects from the same population as Experiment 1 were recruited to participate in this study (see **Table 1** for participants’ characteristics). They were tested 2 weeks after they had participated in Experiments 1 and 2.

#### Materials and Designs

These were identical to those described in Experiment 1 except that 10 sentence fragments were replaced by new sentence fragments (see Appendix B for materials used in this experiment). Such replacement was done so that participants would feel that they were performing an experiment that used a different task and different materials from Experiment 1.

#### Procedure

Participants were seated in front of a laptop and completed the sentence fragments. Experiment 3 used the same background color cues as in Experiment 1 (see the procedure described in Experiment 1). As in Experiment 1, participants were instructed to use a noun–adjective string to describe the target pictures. The main difference between Experiment 1 and Experiment 3 was that in Experiment 3 participants were told to use only adjectives of noun phrases from the other language in the switch trials. In the non-switch trials they had to describe pictures using both adjectives and nouns from the same language depending on which language was requested. Prior to the experiment, participants were given eight practice trials in order to familiarize themselves with the experimental task. Instructions were given in Persian. Participants were informed that their speech would be recorded.

#### Scoring and Data Analysis

The scoring and data analysis were identical to those described in Experiment 1.

### Results

Overall, 928 sentence fragments consisting of 464 switched and 464 non-switched sentence fragments were completed by the participants. Twenty-eight (3%) of the responses were scored as “other” and removed from the analysis. Then the analysis is based on the remaining 900 sentence fragment completions. In sharp contrast to Experiments 1 and 2 in which the grammar of the other language did not considerably affect participants’ responses, the syntactic feature of the other language significantly affected participants’ responses. The results showed that participants used the adjective placement rule from the other language in (28%) of the responses. Inappropriate responses occurred more frequently in the switch conditions (93%) than in the non-switch conditions (7%). **Table 8** shows participants’ responses per condition. The results demonstrated that in the switch conditions inappropriate responses occurred more frequently in switches from L2 to L1 (65.27%) than in switches from L1 to L2 (36.82%).

**Table 8:** Experiment 3: participants’ responses in switch and non-switch tasks.

	Responses			
Language task	Sum	Appropriate	% inappropriate	Omission
Non-switch tasks	464	441	18 (3.87)	5
Persian	232	215	14 (77.77)	3
English	232	226	4 (22.22)	2
Switch tasks	464	202	239 (51.50)	23
Persian-English	232	142	83 (36.82)	7
English-Persian	232	60	156 (65.27)	16

*LT, Language task; Omission: responses scored as other, % inappropriate: the percentage of inappropriate responses (responses scored as “other” were not included).*

As in Experiments 1 and 2, χ^2^-tests were conducted to determine the model of best fit (see **Table 9**). The results indicated that language task (trial type) was highly significant (*p* < 0.001). Adding both language task and target language as predictors improved the model significantly. Then target language affects the responses when language task is taken into account. When language task is removed from the data analysis, there was a significant interaction between target language and source language (*p* < 0.001) in Experiment 3.

**Table 9:** Models of responses in Experiment 3.

Predictor	Estimate	*SE*	*z*-value	*p*
Language task as main predictor: χ^2^(1) = 48.51, *p* < 0.001, *N* = 900				
(Intercept)	-7.384	0.734	-10.060	<0.001
Language task	3.811	0.420	9.078	<0.001
Language task and target language as predictors: χ^2^(1) = 83.747, *p* < 0.001, *N* = 900				
(Intercept)	-4.820	0.538	-8.963	<0.001
Language task	3.830	0.284	13.507	<0.001
Target language	-1.737	0.201	-8.630	<0.001

As in Experiments 1 and 2, language proficiency was tested in interaction with experimental predictors. No significance of English proficiency × source language, proficiency × target language, self-rated speaking proficiency × source language, and self-rated listening proficiency × source language interaction was observed in Experiment 3. The results revealed that the language × condition interaction was significant (*p* > 0.4). The rated self-rated speaking proficiency × target language interaction was significant (*p* < .002). Target language × self-rated speaking proficiency is model of best fit.

The results clearly indicate that participants may inhibit the syntactic properties of one language and use the syntactic feature from the other language.

### Discussion

When participants were asked to describe pictures using both a noun and an adjective from the same language or from the other language in the switch and non-switch trials respectively (see Experiments 1 and 2), they used the correct adjective placement feature of the intended languages in most cases. But in Experiment 3, when they were asked to use only the adjectives of the NP structures from the other language, participants were considerably blind to their uses of the combinatorial nodes (adjective placement rule), suggesting that in Experiment 3, adjectives had much less syntactic restrictions to find their positions in noun phrase structures compared to Experiments 1 and 2. That is, participants’ choices of the combinatorial nodes of adjectives were more volatile in Experiment 3 compared to Experiments 1 and 2. Using syntactic features from the non-target language was stronger under some linguistic contexts than the others. While language task had a significant effect on participants’ responses, with the exception of the interaction of self-rated speaking proficiency × target language, no significant effect of language proficiency on cross-linguistic influence was observed.

## General Discussion

The main aim of the present study was to examine whether there are any cases in which an inherent syntactic feature of a lexical item is inhibited, and the syntactic feature that belongs to the other language is used, instead. It was hypothesized that since the two languages of bilingual speakers are co-activated during language production, the grammatical system of the non-target language may affect the production of the target language (see Schwartz and Kroll, 2006). The results, especially from Experiment 3, confirm the main hypothesis of the study. The results showed interference with respect to combinatorial processing. However, cross-linguistic influences affected differentially by whether only adjectives were switched or both the nouns and adjectives of noun phrases were switched. In Experiments 1 and 2, adjectives sometimes used the syntactic feature (i.e., the combinatorial node) from the other language. In Experiment 3, however, participants used the adjective placement rule from the other language more frequently.

It was also hypothesized that more inappropriate responses are made in the switch tasks than in the non-switch tasks. The results indicated that in all experiments, inappropriate responses occurred more frequently in the switch conditions than in the non-switch conditions.

The results of the experiments, especially Experiment 3, demonstrated interference between bilinguals’ two language systems during speech production. The results indicated that both languages are co-activated in bilingual language production and that bilingual speakers may use the grammar of one language and the word from the other language. The results are consistent with Nicoladis’ (2006) study. She examined whether overlap/ambiguity of adjective–noun strings in English and French leads to transfer. In her study, French-English preschool bilingual children named pictures using an adjective–noun string. Their responses were compared to English and French monolingual children. The results of the study showed that bilinguals made more reversals of pre-nominal French adjectives (e.g., “une personne grand” lit. “a person big”) than monolingual peers. Moreover, they reversed more post-nominal adjectives (e.g., “un ray’e dinosaure” lit. “a striped dinosaur”) than monolingual children. However, more adjective reversal occurred in French, because French uses two adjective–noun orders. The researcher views cross-linguistic transfer as “an epiphenomenon of speech production” (p. 26).

In all three experiments participants used an adjective–noun string to describe pictures; however, their language production differed with respect to combinatorial processing in the three experiments. One of the main aims of the study was to discuss what might cause such differences, and what implications do the results of the present study have for language processing in bilingual speakers. I suggest that in the present study, different experimental contexts led to different patterns of control mechanism in bilingual language processing, because as Green (2011) states, differences in experimental contexts lead to differences in neural loci at which lexical items from the target language can be selected. Accordingly, all “speakers adjust their behavior during an experiment to the specific control demands it imposes” (Green and Abutalebi, 2013, p. 522). Different experimental contexts and the external instructions given to participants may lead to changes in the strength between the nodes within the network, suggesting that exogenous factors may affect the control mechanism (Green, 2011). To put it differently, the pattern of strength between the nodes (e.g., a lemma node and its corresponding combinatorial node) may vary depending on the context in which languages are used. Consequently, the changes in the strength between the nodes may yield in different linguistic behavior.

Now I consider how the results of the present study may be integrated with Hartsuiker and Pickering’s (2008) integrated model of syntactic representation. Below an outline of the model is given first, followed by a description of the results using a model of adjective-head noun/head noun-adjective in bilingual sentence production. In the switch trials in Experiments 1 and 2, participants used both a noun and an adjective from the other language. In the non-switch trials, however, a noun and an adjective had to be selected from the base language. In the switch trials in Experiment 3, participants used only the adjective of the noun-adjective string from the other language. Thus, what is common in all experiments is that producing responses involves activating the appropriate noun lemma together with (a) its category information (noun), (b) its featural information (e.g., singular/plural), (c) the language node (e.g., Persian) and activating the appropriate adjective lemma together with (a) its category information (adjective), (b) its combinatorial information (prenominal/post-nominal), and (c) the language node (e.g., Persian). According to the model, when a Persian-English bilingual speaker intends to produce “pirāhan siāh” (lit. “shirt black”), the concept of “PIRAHAN SIAH” sends activation to the Persian lemma “pirāhan”(shirt) and “siāh”(black). Since the concept is shared between the two languages (Hartsuiker and Pickering, 2008), it also sends activation to the English lemmas (i.e., “black” and “shirt”) to a lesser degree (see Schoonbaert et al., 2007).

**FIGURE 1:**
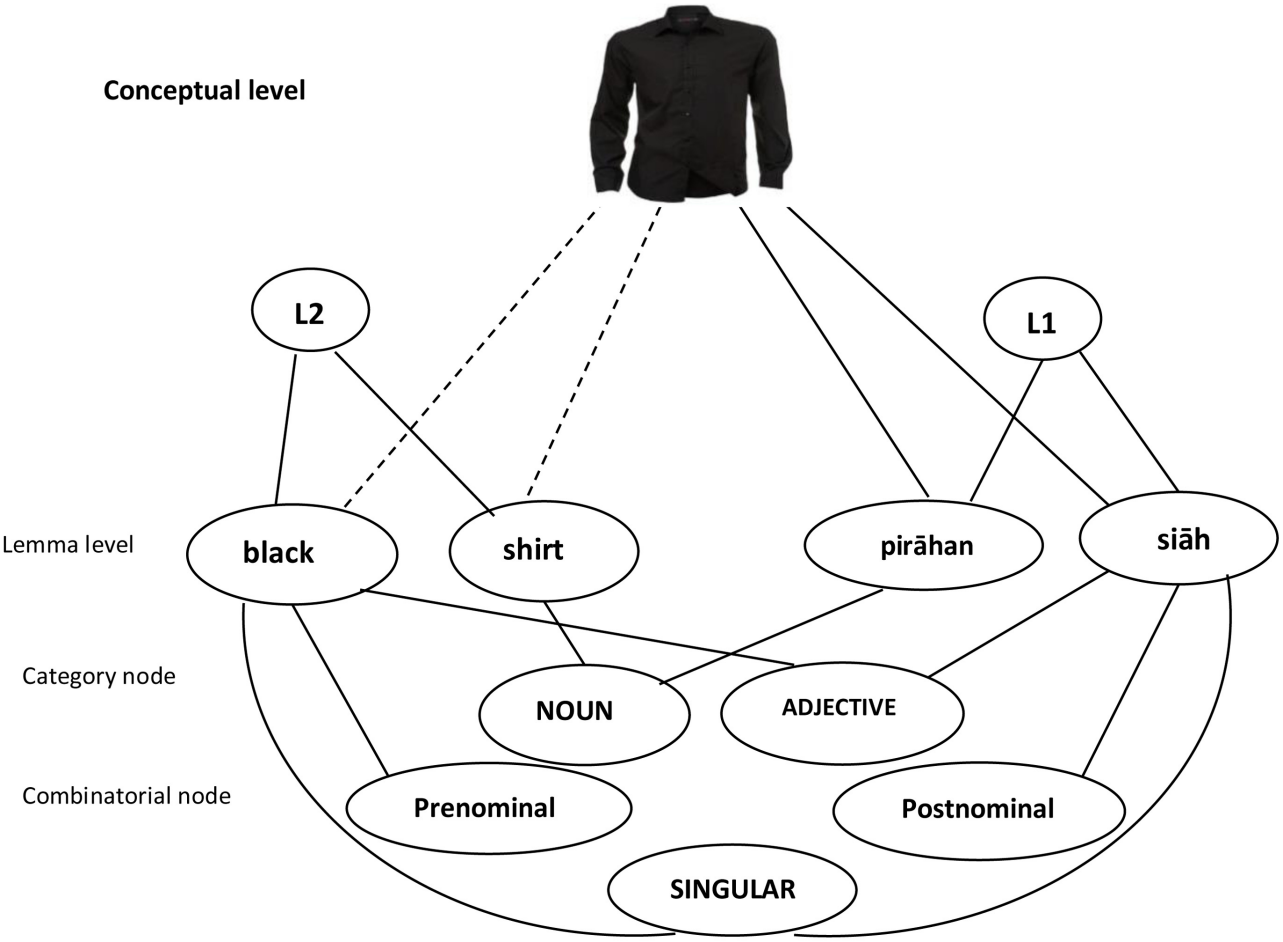
**A model of adjective-head noun/head noun-adjective in bilingual sentence production integrated with Hartsuiker and Pickering’s (2008) integrated model of syntactic representation.** The concept of “Pirāhan siāh” sends activation to the Persian lemma “Pirāhan and “siāh.” The concept also sends activation to the English lemmas, “black” and “shirt” to a lesser degree. “Siāh” is linked to the Persian node (L1), the conceptual node “SIāH,” the adjective node and the post-nominal node. “Black” is linked to the English node (L2), the conceptual node “BLACK,” the adjective node, and the prenominal node. Both “Pirāhan” and “shirt” are linked to the same category node (noun) and featural node (singular).

According to the model, “siāh” is linked to the Persian node (L1), the conceptual node “SIĀH,” the adjective node, and the post-nominal node. “Black” is linked to the English node (L2), the conceptual node “BLACK,” the adjective node, and the prenominal node (see **Figure 1**). Both “Pirāhan” and “shirt” are linked to the same category node (Noun). As stated above, when a Persian-English bilingual speaker intends to produce “siāh,” first the conceptual node “SIĀH” is activated. Then activation spreads to the “siāh” lemma, the Persian language node, and the post-nominal node (combinatorial node). According to the model, the “SIĀH” conceptual node activates the “black” lemma as well, but since the “black” lemma receives little support from the language node (Persian), activation of the lemma “black”-belonging to the other language- is weaker (see Nicoladis, 2006). But even the little activation of the “black” lemma leads to the activation of the prenominal node to a lesser degree (Hatzidaki et al., 2011). In other words, while a Persian-English bilingual speaker normally uses the “siāh” adjective following a noun (i.e., he or she uses the post-nominal combinatorial node), sometimes he or she uses “siāh” before a noun (i.e., he or she uses the prenominal combinatorial node).

The results suggest that producing a Persian adjective, for instance, “derāz (long)” in a construction such as “xatkeš-e derāz” (lit. “ruler long”), causes the activation of the lemma node “derāz,” the NA combinatorial node, the link between the lemma node (derāz) and the combinatorial node, and the category node (adjective). The combinatorial node retains activation at least temporarily (cf. Branigan et al., 1999), and a bilingual speaker is more likely to use the same combinatorial node (post-nominal) again even when using an English adjective (Cleland and Pickering, 2003). In other words, the concurrent activation of an NA combinatorial node might “lead to the strengthening of the link between the lemma nodes” (p. 217) in the other language of a bilingual (here English) and the NA combinatorial node. Cleland and Pickering (2003) suggested that “more generally, the activation of combinatorial nodes is related to the construction of constituent-structure representations” (p. 216). Accordingly, an NA combinatorial node is activated when a Persian adjective (e.g., qermez, meaning red) is used in the noun-adjective construction. In the same vein, an AN combinatorial node is activated when an English adjective (e.g., green) is used in the adjective–noun string (see Pickering and Branigan, 1998). Accordingly, producing an English construction involving an adjectival construction such as “long road” involves the prenominal adjectival modification, whereas producing a Persian NP such as “jāddeh-ye tulāni” (lit. “road long”) involves the post-nominal adjectival modification. Thus, the constructions are associated with a combinatorial node, A,N and N,A nodes, respectively (Cleland and Pickering, 2003).

**FIGURE 2:**
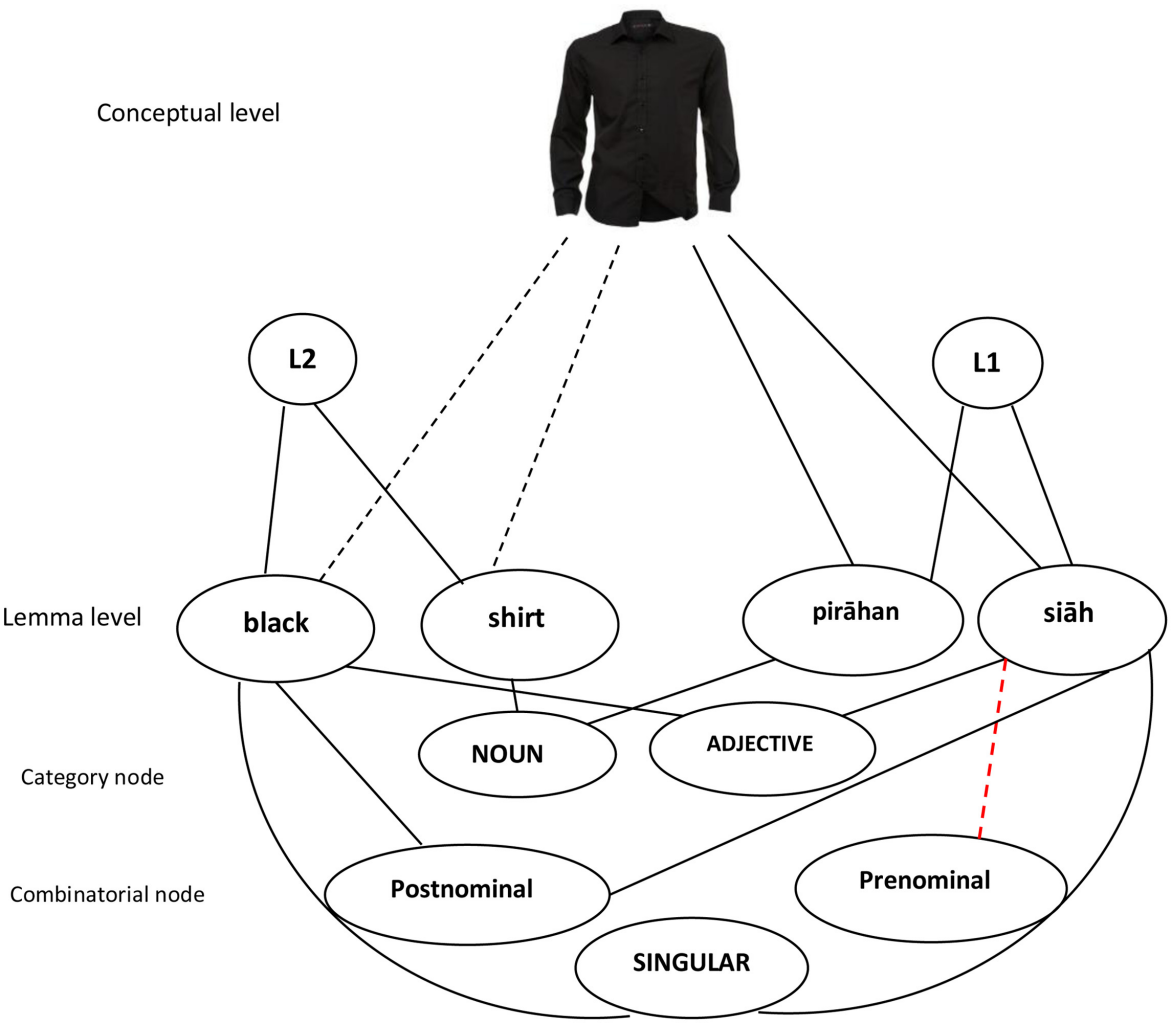
**A model of syntactic interference inside NP structure integrated with Hartsuiker and Pickering’s (2008) integrated model of syntactic representation.** The dotted red line shows the temporary link between a Persian adjective with the combinatorial node of the other language (English).

As stated above, since the link between a lemma node and a certain combinatorial node retains activated, it is more likely that the same link is used between a lemma node from the other language and the activated combinatorial node in the subsequent production of an adjective–noun string. This may explain why a Persian-English bilingual produces “mard-e old” (lit. “man old”) after he/she produces a NA construction such as “māhi-e bozorg” lit. “fish big.” Bilinguals’ switching back and forth between the two languages has a critical role in increasing the activation of the non-target language lemmas and the syntactic information (i.e., featural and combinatorial information) associating with them. In the adjective case, this leads to using the combinatorial node from the other language (see **Figure 2**).

The results showed that 65, 80, and 78% of the responses in Experiments 1–3, respectively, in which participants used the combinatorial node from the other language occurred after they used the same combinatorial node in the previous trail. The results demonstrated that participants had the tendency to produce sentences with the syntactic structure of a self-produced sentence during language production. Given this situation, producing a construction that employs a NA construction (e.g., “pesar-e mariz” lit. “boy sick”) enhances the likelihood of producing the subsequent construction using the NA structure. This occurs because “combinatorial nodes retain activation after use” (Cleland and Pickering, 2003, p. 217).

There is a debate about whether grammatical feature selection is an automatic consequence of lexical node selection (see Schiller and Caramazza, 2003). Caramazza (1997) distinguishes between “intrinsic” grammatical features and “extrinsic” grammatical features. Intrinsic grammatical features are considered as inherent features of lexical items, however, extrinsic grammatical features are those features that “are not inherently associated with a word and are determined contextually (e.g., number, tense)” (Purmohammad, 2015, p. 88). Whereas ‘gender’ is considered as an arbitrary property, ‘verb’ is not an arbitrary feature of a lexical item. He suggests that the accessibility of different grammatical features is not uniform (Caramazza, 1997). Accordingly, while gender features are not automatically activated by the semantic network, tense and grammatical class (e.g., noun) features “do receive activation from the semantic network” (p. 195). I suggest that as the combinatorial feature is an inherent feature of a lexical item, it is automatically activated. If this were the case, the question arises how we can account for cases where a word uses the combinatorial feature from the other language? According to the models that posit that inherent grammatical features are automatically activated (see Caramazza, 1997; Levelt et al., 1999) when an adjective (a lexical node) is activated, its combinatorial node (prenominal or post-nominal position) is automatically activated. If this were the case, I suggest that when an adjective is linked to the combinatorial node that belongs to the other language, its activated combinatorial node is suppressed (deactivated) and the syntactic feature from the other language is retrieved, instead. Thus, an additional locally control (i.e., a local reactive inhibition) is exerted in order to inhibit the activated syntactic feature (see Colzato et al., 2008 for the term “reactive”). If the Caramazza’s (1997) account that when a lexical node it activated, its inherent grammatical features (e.g., the combinatorial node) are automatically activated were not the case, an alternative interpretation would be that a lexical node is directly linked to the combinatorial node that belongs to the other language without the need to suppress the word’s intrinsic syntactic feature.

The results may also be interpreted in terms of the asymmetric switching cost account (see Meuter and Allport, 1999). In the present study (78, 89, and 65%) of the inappropriate responses in Experiments 1–3, respectively, occurred in switches from L2 to L1. The results are consistent with Meuter’s (1994) and Meuter and Allport (1999) study. Meuter and Allport (1999) reported that when a bilingual speaker switches, the cost of switching (reaction time) is greater when he switches from his L2 to his L1 than vice versa. In other words, switching in bilingual language production follows from asymmetric switching costs. The asymmetric switching cost account postulates that in code-switched utterances when the intended response language is participants’ L1, we expect stronger recording of the distractor (see Meuter, 2005). Moreover, we expect more inappropriate responses when the intended response language is participants’ L1. The results of the study are in line with Meuter (1994) and Meuter and Allport (1999) in that more responses (59%) scored as “other” occurred in switches from L2 to L1 suggesting that switches from L2 to L1 are more costly than vice versa. Participants had more difficulty making appropriate responses in switches from L2 to L1 than from L1 to L2, because bilingual speakers experience much more difficulty when they have to “suppress a resulting inappropriate response” (Meuter, 2005, p. 355) in their L1. According to Meuter and Allport (1999) the reason for the paradoxical pattern in the switch conditions is that the inhibition of L1 is considerably powerful in non-balanced bilingual speakers. Thus, the cost that arises from its removal is considerably large (see Green, 1993, 1998). To connect the Hartsuiker et al. (2004) model of syntactic representation model with Meuter and Allport’s (1999) findings, the results of the present study reveal that participants had more difficulty reactivating the combinatorial node (prenominal) of Persian when switching from L2 to L1. This yielded in more inappropriate responses in switches in this direction. Accordingly, the reason why less inappropriate responses were observed in switches from L1 to L2 may be that speaking in L1 requires little active inhibition of L2 (Meuter and Allport, 1999), therefore, in L1 to L2 switches participants needed less effort to reactivate their L2. Moreover, I interpreted the results of the present study in terms of the inhibitory processes (see Green, 1986, 1998). The presence of asymmetric language switch pattern is viewed as the main evidence supporting the use of inhibitory process (see Meuter and Allport, 1999; Costa and Santesteban, 2004). Thus, the results are in favor of the inhibition process in bilingual language production.

## Conclusion

The results indicated that bilingual speakers may use a word from one language and the grammar from the other language. During bilingual language processing, the syntactic feature of a lexical item may undergo a local reactive inhibition and lexical items may use the syntactic feature from the other language, instead. As a combinatorial node of an adjective “retains activation at least temporarily” (Cleland and Pickering, 2003, p. 217), bilinguals are more likely to use the same combinatorial node again even when producing an adjective from the other language. The findings of the present study keep in line with the interference accounts of syntactic processing in bilinguals’ language production, and the parallel activation of the two languages during speech production. More syntactic interference occurred in the switch tasks in which the two languages of a bilingual speaker were involved to a greater degree. Most of the inappropriate responses were produced in switches from L2 to L1 than from L1 to L2. While language proficiency did not put effects on responses, language task and target language significantly affected participants’ responses.

## Conflict of Interest Statement

The author declares that the research was conducted in the absence of any commercial or financial relationships that could be construed as a potential conflict of interest.

## References

[B1] Aguirre A. (1976). *Acceptability Judgments of Code-Switching Phrases by Chicanos: Some Preliminary Findings*, ed. ERIC. New York, NY: ERIC, 122 129.

[B2] Arias R. Lakshmanan U. (2005). “Code-switching in a Spanish-English bilingual child: a communication resource?,” in *ISB4: Proceedings of the 4th International Symposium on Bilingualism*, eds Cohen J. McAlister K. T. Rolstad K. MacSwan J. (Somerville, MA: Cascadilla Press), 94–109.

[B3] Belazi H. Rubin E. Toribio A. J. (1994). Code switching and X-bar theory: the functional head constraint. *Linguist. Inq.* 25 221–238.

[B4] Bernolet S. Hartsuiker R. J. Pickering M. J. (2007). Shared syntactic representations in bilinguals: evidence for the role of word-order repetition. *J. Exp. Psychol. Learn. Mem. Cogn.* 33 931–949. 17723070 10.1037/0278-7393.33.5.931

[B5] Branigan H. P. Pickering M. J. Cleland A. A. (1999). Syntactic priming in written production: evidence for rapid decay. *Psychon. Bull. Rev.* 6 635–640. 10.3758/BF03212972 10682206

[B6] Broersma M. (2011). “Triggered code-switching: evidence from picture naming experiments,” in *Modeling Bilingualism from Structure to Chaos: In Honor of Kees de Bot*, eds Schmid M. S. Lowie W. (Amsterdam: John Benjamins), 37–57.

[B7] Bullock B. E. Toribio A. J. (2009). “Themes in the study of code-switching,” in *The Cambridge Handbook of Linguistic Code-Switching*, eds Bullock B. E. Toribio A. J. (Cambridge: Cambridge University Press), 1–17.

[B8] Cantone K. F. MacSwan J. (2009). “Adjectives and word order: a focus on Italian-German codeswitching,” in *Multidisciplinary Approaches to Code Switching*, eds Isurin L. Winford D. de Bot K. (Amsterdam: John Benjamins Publishing Company), 243–278.

[B9] Caramazza A. (1997). How many levels of processing are there in lexical access? *Cogn. Neuropsychol.* 14 177–208. 10.1080/026432997381664

[B10] Cleland A. A. Pickering M. J. (2003). The use of lexical and syntactic information in language production: Evidence from the priming of noun-phrase structure. *J. Mem. Lang.* 49 214–230. 10.1016/S0749-596X(03)00060-3

[B11] Colzato L. S. Bajo M. T. van den Wildenberg W. Paolieri D. Nieuwenhuis S. La Heij W. (2008). How does bilingualism improve executive control? A comparison of active and reactive inhibition mechanisms. *J. Exp. Psychol. Learn. Mem. Cogn.* 34 302–312. 10.1037/0278-7393.34.2.302 18315407

[B12] Costa A. (2005). “Lexical access in bilingual production,” in *The Handbook of Bilingualism. Psycholinguistics Approaches*, eds Kroll J. F. De Groot A. M. B. (New York, NY: Oxford University Press), 309–325.

[B13] Costa A. Roelstraete B. Hartsuiker R. J. (2006). The lexical bias effect in bilingual speech production: evidence for feedback between lexical and sublexical levels across languages. *Psychon. Bull. Rev.* 13 972–977. 10.3758/BF03213911 17484421

[B14] Costa A. Santesteban M. (2004). Lexical access in bilingual speech production: evidence from language switching in highly proficient bilinguals and L2 learners. *J. Mem. Lang.* 49 491–511. 10.1016/j.jml.2004.02.002

[B15] de Bot K. (1992). A bilingual production model: levelt’s ‘Speaking’ model adapted. *Appl. Linguist.* 13 1–24.

[B16] de Bot K. Schreuder R. (1993). “Word production and the bilingual lexicon,” in *The Bilingual Lexicon*, eds Schreuder R. Weltens B. (Amsterdam: Benjamins), 191–214.

[B17] Francis W. S. (2005). “Bilingual semantic and conceptual representation,” in *Handbook of Bilingualism: Psycholinguistic Approaches*, eds Kroll J. F. de Groot A. M. (New York, NY: Oxford University Press), 251–268.

[B18] Gil L. A. Eichler N. Jansen V. Patuto M. Müller N. (2012). “The syntax of mixed DPs containing an adjective: evidence from bilingual german-romance (French, Italian, Spanish) children,” in *Proceedings of the 14th Hispanic Linguistics Symposium*, eds Geeslin K. Díaz-Campos M. (Somerville, MA: Cascadilla Proceedings Project), 242–257.

[B19] Green D. W. (1986). Control, activation and resource. *Brain Lang.* 27 210–223. 10.1016/0093-934X(86)90016-7 2420411

[B20] Green D. W. (1993). “Towards a model of L2 comprehension and production,” in *The Bilingual Lexicon*, eds Schreuder R. Weltens B. (Amsterdam: John Benjamins Publishing Company), 249–277.

[B21] Green D. W. (1998). Mental control of the bilingual lexico-semantic system. *Biling. Lang. Cogn.* 1 67–81. 10.1017/S1366728998000133

[B22] Green D. W. (2011). Language control in different contexts: the behavioural ecology of bilingual speakers. *Front. Psychol.* 2:103 10.3389/fpsyg.2011.00103 PMC3132677 21779260

[B23] Green D. W. Abutalebi J. (2013). Language control in bilinguals: the adaptive control hypothesis. *J. Cogn. Psychol.* 25 515–530. 10.1080/20445911.2013.796377 PMC4095950 25077013

[B24] Grosjean F. (2008). *Studying Bilinguals.* Oxford: Oxford University Press.

[B25] Hamers J. F. Blanc M. H. A. (1989). *Bilinguality and Bilingualism*, Revised edn, Cambridge: Cambridge University Press.

[B26] Hartsuiker R. J. Pickering M. J. (2008). Language integration in bilingual sentence production. *Acta Psychol.* 128 479–489. 10.1016/j.actpsy.2007.08.005 17870040

[B27] Hartsuiker R. J. Pickering M. J. Veltkamp E. (2004). Is syntax separate or shared between languages? Cross-linguistic syntactic priming in Spanish-English bilinguals. *Psychol. Sci.* 15 409–414. 10.1111/j.0956-7976.2004.00693.x 15147495

[B28] Hatzidaki A. Branigan H. Pickering M. J. (2011). Co-activation of syntax in bilingual language production. *Cogn. Psychol.* 62 123–150. 10.1016/j.cogpsych.2010.10.002 21093856

[B29] Karousou-Fokas R. Garman M. (2001). Psycholinguistic interpretation of codeswitching: evidence from fluent Greek–English bilingual adults. *Int. J. Biling.* 1 39–69. 10.1177/13670069010050010301

[B30] Kootstra G. J. van Hell J. G. Dijkstra T. (2010). Syntactic alignment and shared word order in code-switched sentence production: evidence from bilingual monologue and dialogue. *J. Mem. Lang.* 63 210–231. 10.1016/j.jml.2010.03.006

[B31] Kroll J. F. Bobb S. C. Misra M. Gue T. (2008). Language selection in bilingual speech: evidence for inhibitory process. *Acta Psychol.* 128 416–430. 10.1016/j.actpsy.2008.02.001 PMC2585366 18358449

[B32] Kroll J. F. Bobb S. C. Wodnecka Z. (2006). Language selectivity is the exception, not the role: arguments against a fixed locus of language selection in bilingual speech. *Lang. Cogn.* 9 119–135. 10.1017/S1366728906002483

[B33] La Heij W. (2005). “Selection processes in monolingual and bilingual lexical access,” in *Handbook of Bilingualism: Psycholinguistic Approaches*, eds Kroll J. F. de Groot A. M. (New York, NY: Oxford University Press), 289–307.

[B34] Levelt W. J. M. Roelofs A. Meyer A. S. (1999). A theory of lexical access in speech production. *Behav. Brain Sci.* 22 1–75. 10.1017/S0140525X99001776 11301520

[B35] MacSwan J. (2009). “Generative approaches to code-switching,” in *The Cambridge Handbook of Linguistic Code-Switching*, eds Bullock B. E. Toribio A. J. (Cambridge: Cambridge University Press), 309–335.

[B36] Mahootian S. (2006). “Code switching and mixing,” in *Encyclopedia of Language and Linguistics*, 2nd Edn, ed. Brown K. (Oxford: Elsevier).

[B37] McClure E. (1981). “Formal and functional aspects of the codeswitched discourse of bilingual children,” in *Latino Language and Communication Behavior*, ed. Duran R. P. (Norwood, NJ: ABLEX Publishing Corporation), 69–94.

[B38] McClure E. (1977). “Aspects of code-switching in the discourse of bilingual Mexican-American children,” in *Georgetown University Round Table*, ed. Saville-Troike M. (Washington, DC: Georgetown University Press).

[B39] Meuter R. F. I. (1994). *Language Switching in Naming Tasks.* Oxford: University of Oxford.

[B40] Meuter R. F. I. (2005). “Language selection in bilinguals: mechanisms and processes,” in *Handbook of Bilingualism: Psycholinguistic Approaches*, eds Kroll J. F. de Groot A. M. (New York, NY: Oxford University Press), 349–370.

[B41] Meuter R. F. I. Allport A. (1999). Bilingual language switching in naming: asymmetrical costs of language selection. *J. Mem. Lang.* 40 25–40. 10.1037/a0034060

[B42] Myers-Scotton C. (1993). *Duelling Languages: Grammatical Structure in Codeswitching*. Oxford: Clarendon Press.

[B43] Myers-Scotton C. (2002). *Contact Linguistics: Bilingual Encounters and Grammatical Outcomes*. New York, NY: Oxford University Press.

[B44] Nartey J. (1982). Code-switching, interference or faddism? Language use among educated Ghanians. *Anthropol. Linguist.* 24 183–192.

[B45] Nicoladis E. (2006). Cross-linguistic transfer in adjective-noun strings by preschool bilingual children. *Biling. Lang. Cogn.* 9 15–32. 10.1017/S136672890500235X

[B46] Pandit I. (1990). “Grammaticality in code switching,” in *Codeswitching as a Worldwide Phenomenon*, ed. Jacobson R. (New York, NY: Peter Lang), 33–69.

[B47] Paradis M. (1993). Linguistic, psycholinguistic, and neurolinguistic aspects of interference in bilingual speakers: the activation threshold hypotheses. *Int. J. Psychol.* 9 133–145.

[B48] Pfaff C. W. (1979). Constraints on language mixing: intrasentential codeswitching and borrowing in Spanish/English. *Language* 55 291–318. 10.2307/412586

[B49] Pickering M. J. Branigan H. P. (1998). The representation of verbs: evidence from syntactic priming in language production. *J. Mem. Lang.* 39 633–651. 10.1006/jmla.1998.2592

[B50] Poplack S. (1980). Sometimes I’ll start a sentence in Spanish y termino en espanol: towards a typology of codeswitching. *Linguistics* 18 581–618. 10.1515/ling.1980.18.7-8.581

[B51] Purmohammad M. (2015). *Codeswitching: A Touchstone of Models of Bilingual Language Production*. Ph.D. thesis, University of Bern. Switzerland.

[B52] Sankoff D. Poplack S. (1981). A formal grammar for codeswitching. *Papers Linguist.* 14 3–46. 10.1080/08351818109370523

[B53] Schiller N. O. Caramazza A. (2003). Grammatical feature selection in noun phrase production: evidence from German and Dutch. *J. Mem. Lang.* 48 169–194. 10.1016/S0749-596X(02)00508-9

[B54] Schoonbaert S. Hartsuiker R. J. Pickering M. J. (2007). The representation of lexical and syntactic information in bilinguals: evidence from syntactic priming. *J. Mem. Lang.* 56 153–171. 10.1016/j.jml.2006.10.002

[B55] Schwartz A. I. Kroll J. F. (2006). “Language processing in bilingual speakers,” in *Handbook of Psycholinguistics*, 2nd Edn, eds Traxler M. J. Gernsbacher M. A. (Amsterdam: Elsevier), 967–999.

[B56] Selles A. (2011). *Cross-Linguistic Interference in the Production of Adjective and Noun Word Order by Spanish-English Bilinguals*. Master’s theses, University of Edinburgh, Edinburgh.

[B57] Sobin N. J. (1984). On code-switching inside NP. *Appl. Psychol.* 5 293–303. 10.1017/S0142716400005191

[B58] Voga M. Grainger J. (2007). Cognate status and cross-script translation priming. *Mem. Cogn.* 35 938–952. 10.3758/BF03193467 17910178

