# Supplementary material for: Grammatical Encoding in Bilingual Language Production: A Focus on Code-switching
Source: Front Psychol. 2015 Nov 26;6:1797. doi: 10.3389/fpsyg.2015.01797 (PMC4659875; doi:10.3389/fpsyg.2015.01797)
Supplement: Supplementary file 1 [file Data_Sheet_1.DOCX]

**APPENDIX A: List of items used in Experiment 2**

| Mr Green met a last night  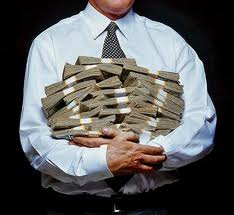 | **red shoes**  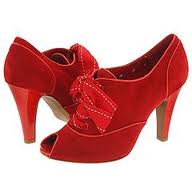  She bought the yesterday. |
| --- | --- |
| **small bucket**  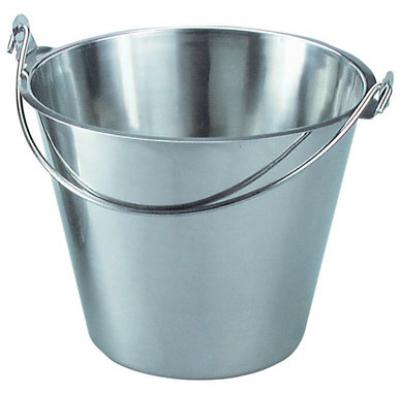  Sherry filled the with water | 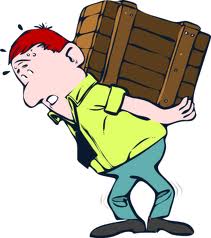  بالاخره تونست اون رو جابجا کنه |
| 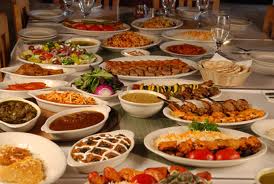  Thank you, We had last night | **tight skirt**  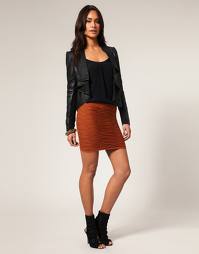  The lady was wearing a at the party |
| **جوراب کثیف**  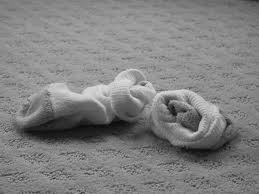  نباید رو روی زمین بندازی | **narrow street**  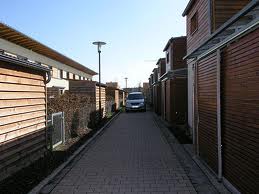  There are very in the little village |
| **hot tea**  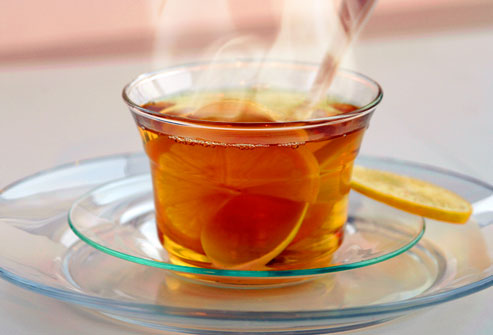  I prefer to water. | **غذای تند**  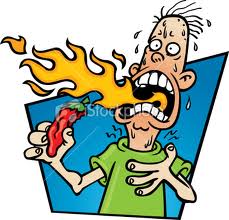  اصلا نمی تونم رو بخورم |
| **expensive necklace**  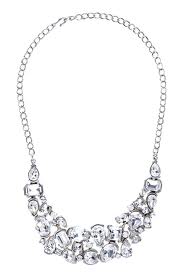  Thomas bought Sarah for her birthday | 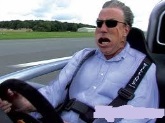  Today the was seen in the street. |
| 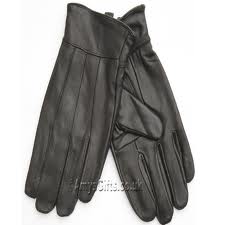 **دستکش چرمی**  امروز میترا رو نپوشید | 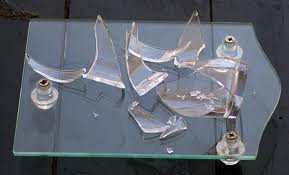  Lily cut herself on the in the kitchen |
| 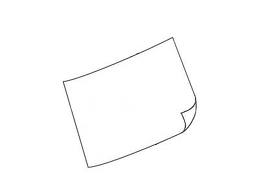 **کاغذ نازک**  نامه را روی یک نوشت | بهمن از روی یه افتاد  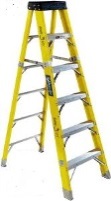 |

| 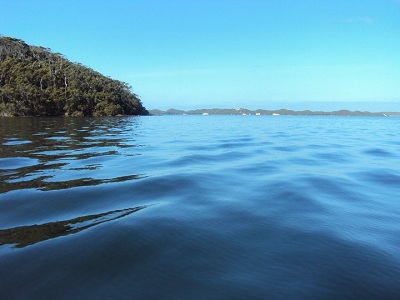  They were swimming in the yesterday | 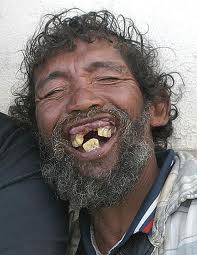 **مرد زشت**  هیچ کی اون رو دوست نداشت |
| --- | --- |
| 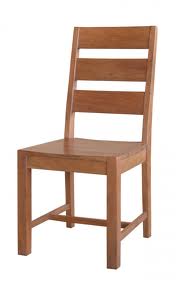  پدربزرگش همیشه روی یه می نشست | 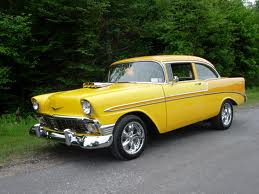  دوست نداره اون رو بفروشه |
| 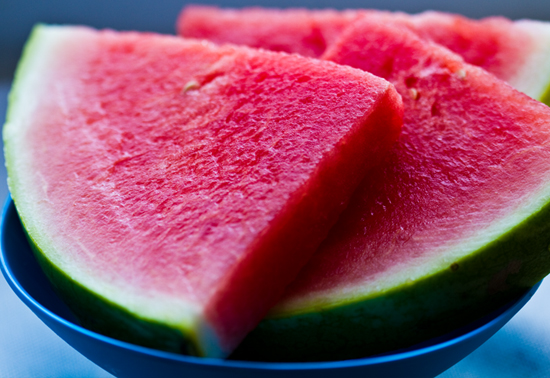  این تو تابستون می چسبه | 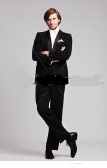 **مرد جوان**  پیر زن دوست داره با یه ازدواج کنه |
| **اتاق تاریک**  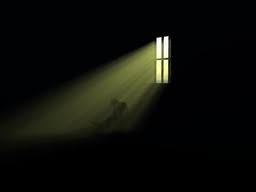  امیر از بدش می آد | 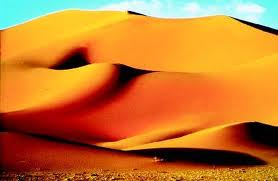  هیچ کس دوست نداره تو این راه بره |
| **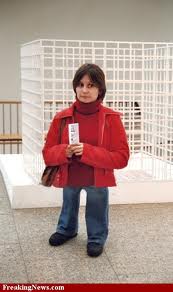 دختر کوتاه قد**  اون روز یه بهش کمک کرد | 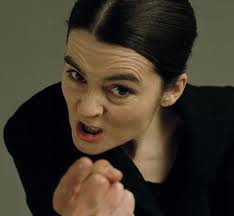  از اون خیلی می ترسه |
| **big spot**  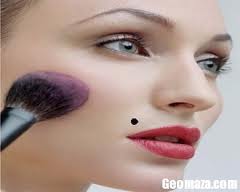  Birdie has got a on her face. | 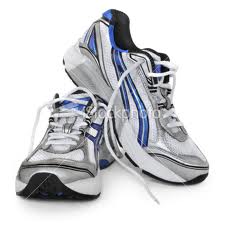  These are not for wedding |
| 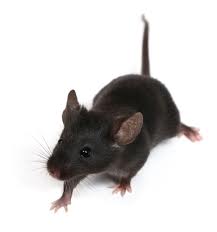  Last night a scurried across the floor | 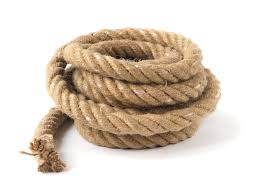  کارگر را محکم گرفت |
| **round table**  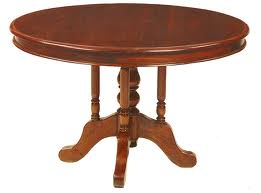  There was a in the dining room | 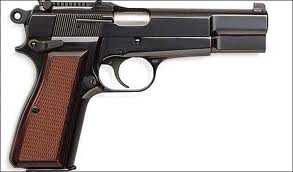  The boy had a in his hand |

**APPENDIX B: Items used in Experiment 3**

| 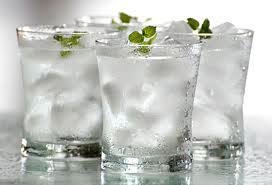  توی تابستون خیلی می چسبه. | 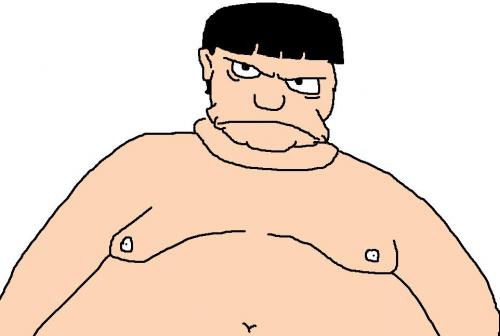  I saw a breaking the door. |
| --- | --- |
| 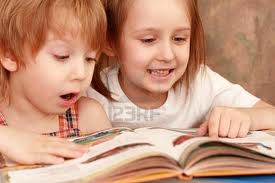  They always read at weekends | 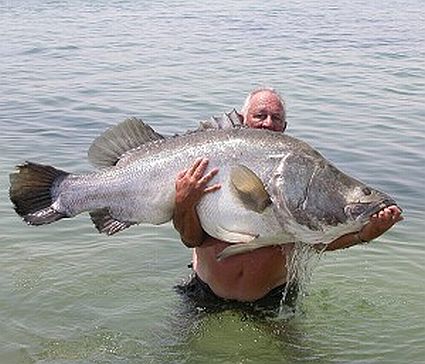  ماهیگیر امسال یه شکار کرد. |
| 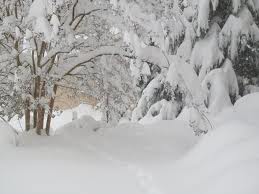  We've had a this year. | 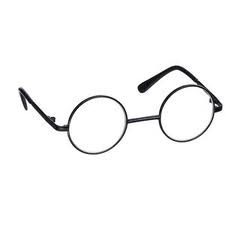  Dale is wearing today. |
| 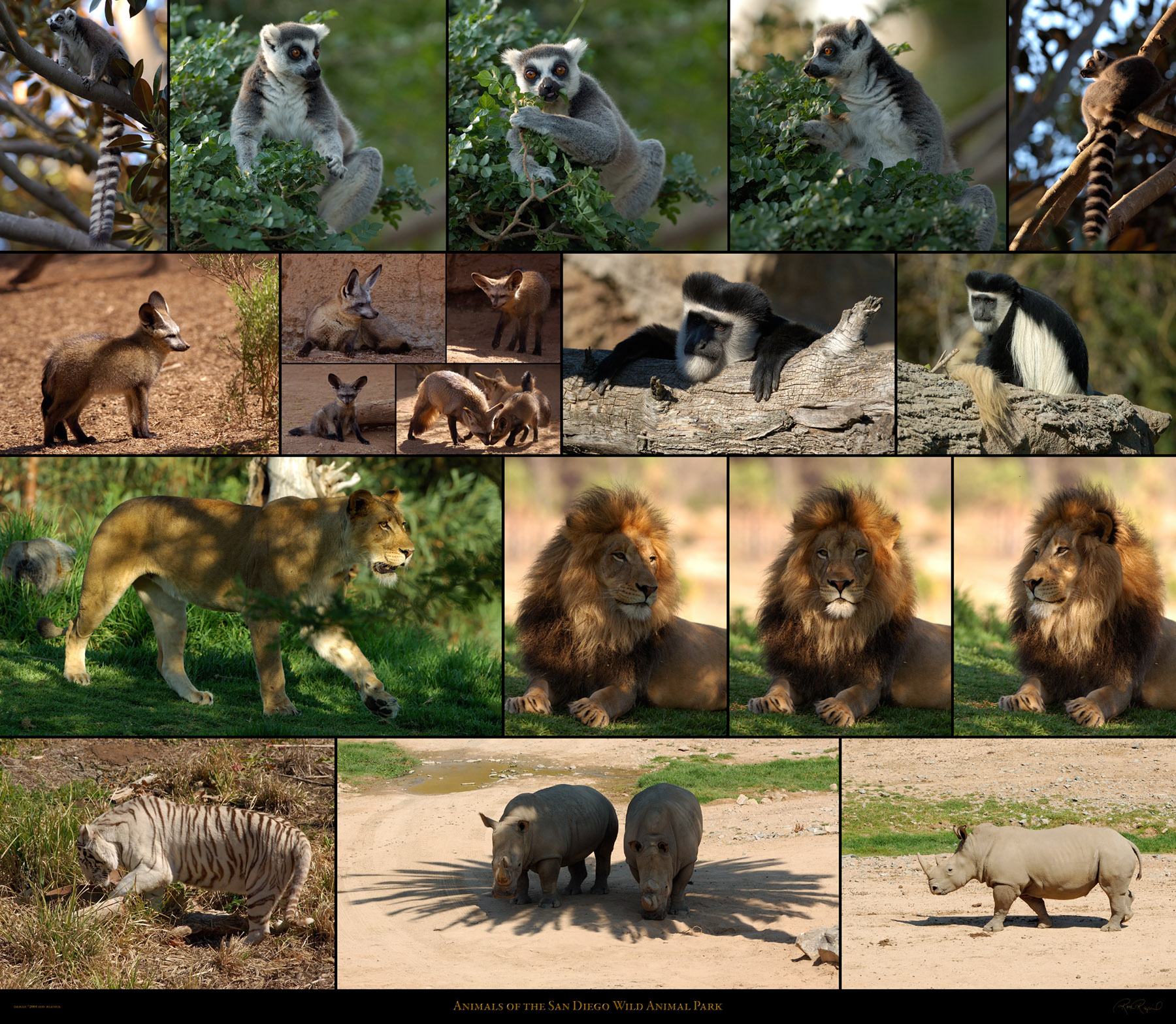  The zoo is full of from Africa. | 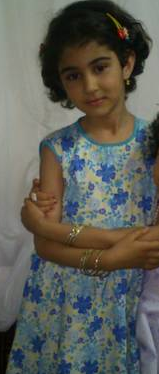  این دختره یه پوشیده |
| 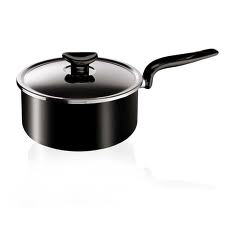  این قابلمه یه داره | 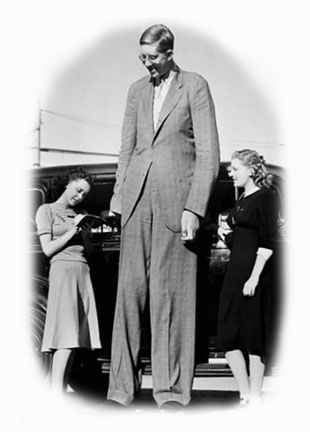  نسرین می خواست اون رو کمک کنه. |
| 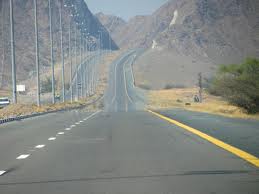  They live on the out of town | 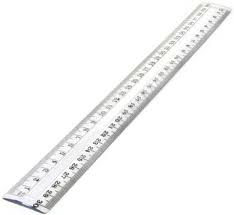  George needs a to rule the line |
| 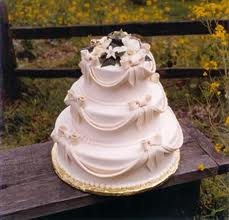  دیشب تو عروسی، خوردیم | 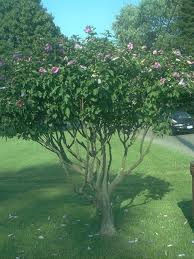  وسط حیاط یه کاشتند |
| 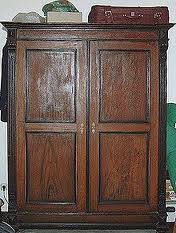  I can’t move the without any help | 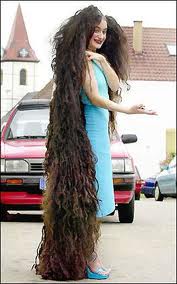  A woman with was walking in the street |

| 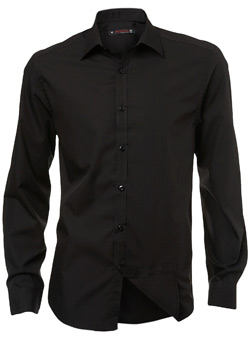  امروز امید یه پوشید | 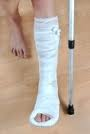  حامد نمی تونست با یه بدود |
| --- | --- |
| 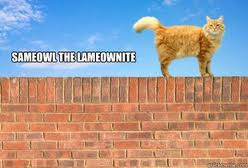  That cat jumped over the quickly. | 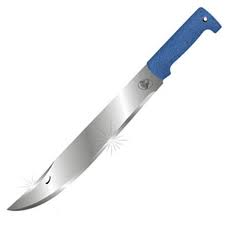  Mariel cut her finger with a in the kitchen |
| 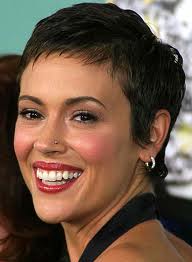  شبنم از آرایشگاه با برگشت | 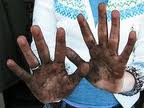  حسین می خواد با آب، ش رو بشوره |
| 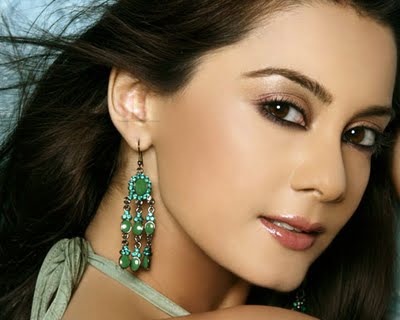  امیر امشب با یه قرار داره | 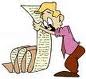  اصلاً وقت ندارم رو بخونم |
| 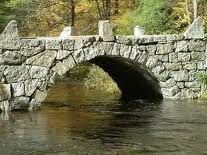  ما باعجله از یه رد شدیم | 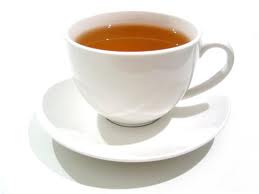  Ted prefers her tea in a and saucer |
| 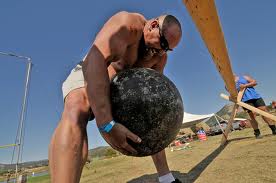  That man could not lift the yesterday | 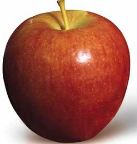  The doctor used to eat every morning |
| 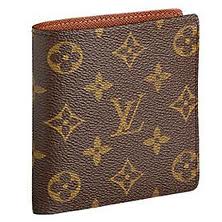  توی این چیزی نمی شه گذاشت | 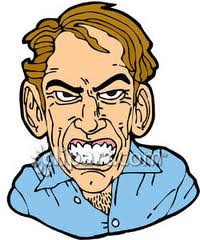  Jack is an isn’t he? |
| 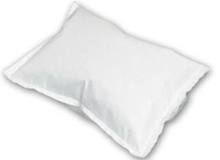  شهرام توی اون اتاق روی یه خوابیده بود | 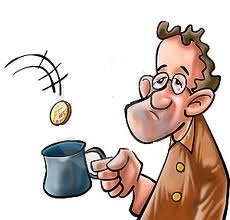  Albert gave change to the in the street |
